# Cardioprotective Chinese herbs and antiretroviral drug metabolism: a systematic review of in vitro evidence

**DOI:** 10.3389/fphar.2025.1627058

**Published:** 2025-11-20

**Authors:** Wan-Jung Cheng, Anthony Jaworowski, Janine M. Trevillyan, Anna C. Hearps, Charlie C. Xue, Anthony L. Zhang

**Affiliations:** 1 School of Health and Biomedical Sciences, Royal Melbourne Institute of Technology (RMIT) University, Bundoora, VIC, Australia; 2 Life Sciences Discipline, Burnet Institute, Melbourne, VIC, Australia; 3 Department of Infectious Diseases, Alfred Hospital, Monash University, Melbourne, VIC, Australia; 4 Department of Infectious Diseases and Immunology, Clinical Virology and HIV Services, Austin Health, Melbourne, VIC, Australia; 5 Department of Infectious Diseases, Peter Doherty Institute of Infection and Immunity, University of Melbourne, Melbourne, VIC, Australia

**Keywords:** Chinese herbal medicine, antiretroviral therapy, antiretroviral drug metabolism, herb–drug interactions, cardiovascular disease, Cytochrome P450 (CYP), *in vitro* assay, people with HIV

## Abstract

**Introduction:**

There is considerable potential for using Chinese Herbal Medicine to manage inflammatory co-morbidities, including cardiovascular disease, in people with HIV. However, any use would require understanding herb–drug interactions with antiretroviral (ARV) drugs to ensure safety. We evaluate evidence for the effect of selected cardioprotective Chinese herbs on the activity of cytochrome P450 (CYP) enzymes that metabolise ARV drugs.

**Methods:**

We conducted a comprehensive review of six Chinese herbs commonly found in Chinese herbal formulas for treating cardiovascular conditions. We examined the effects of their extracts and reference bioactive molecules on CYP expression and enzymatic activity. The review focused on evidence from *in vitro* laboratory studies. The included herbs were Dan Shen, Huang Qi, Bai Zhu, Dang Gui, Chuan Xiong, and Gan Cao. Study quality was assessed using the SciRAP 2.1 risk of bias tool, and results were grouped according to experimental methodology.

**Results:**

426 articles were identified of which 24 met the inclusion criteria. Overall risk of bias was low. Dan Shen and Gan Cao were the most frequently studied herbs. The most common outcome reported was no significant effect on enzyme activity, occurring in 61% of assays for Dan Shen, 37% for Gan Cao, 47% for Dang Gui, and 67% for Huang Qi. Aqueous extracts, representing the most clinically relevant preparation, of Dan Shen had minimal impact on CYP activity, while those of Gan Cao indicated potential for enzyme inhibition. In contrast, aqueous extracts of the other herbs showed a tendency toward enzyme induction.

**Discussion:**

These findings suggest that there is great potential for use of Chinese herbal medicine in managing inflammatory co-morbidities in people with HIV, but that careful consideration of herb-ARV drug interaction is warranted. While Dan Shen appears relatively safe to use in individuals receiving ARV therapy, caution is warranted for other herbs. We highlight the importance of clinically relevant extraction methods in herb–drug interaction studies. Since individual herbs may have opposing effects on ARV drug metabolism, studies conducted using whole formulae are critical.

## 1 Introduction

Antiretroviral (ARV) therapy is the cornerstone for the management of HIV infection, profoundly transforming the prognosis for people with HIV. ARV therapy suppresses viral replication to undetectable levels, thereby preventing the progression to acquired immunodeficiency syndrome (AIDS) and extending the lifespan and quality of life of people with HIV to levels comparable to the general population (Marcus et al., 2020). Despite the remarkable advancements brought by ARV therapy, it cannot eradicate the virus completely from latent reservoirs in the body and consequently must be taken lifelong to prevent viral rebound.

People with HIV receiving suppressive ARV therapy are at an elevated risk of developing comorbid conditions, particularly cardiovascular disease (CVD). A systematic review investigating the prevalence and risk factors of CVD among people with HIV found their CVD risk to be 1.5 to 3 times higher than that of HIV-negative individuals (Ruamtawee et al., 2023). This heightened risk is independent of traditional risk factors and persists despite suppressive ARV therapy, partly attributed to chronic inflammation and immune activation associated with HIV infection (Hsue and Waters, 2019; Jaworowski et al., 2019). The prolonged inflammatory state can accelerate the pathogenesis of CVD, making it a significant concern in the long-term management of HIV.

The presence of comorbidities such as CVD often requires additional medications, significantly increasing the risk of drug–drug interactions. ARV therapy typically involves a combination of two to three drugs, each targeting various stages of the viral life cycle, many of which are metabolized by Cytochrome P450 (CYP) enzymes. This creates a heightened potential for pharmacokinetic interactions, particularly when additional agents—such as herbal medicines—are introduced.

With the rising burden of CVD in people with HIV and limitations in existing treatments, interest in complementary cardiovascular strategies is increasing. Chinese herbal medicine has been used for thousands of years across Asian countries for the prevention and management of CVD, offering a body of traditional knowledge and clinical experience that may complement biomedical approaches.

Although Chinese herbal medicine is recognized as a regulated health profession in some countries, it is generally not included in Western medical education. This lack of awareness among medical practitioners results in limited referrals to Chinese medicine practitioners. Conversely, Chinese medicine practitioners often have minimal training in pharmacology and herb–drug interactions. Bridging this knowledge gap is crucial, and evidence-based research is needed to foster safer, integrative care that combines both Western and Chinese medicine.

To address this critical gap, the current systematic review evaluates *in vitro* studies investigating potential herb-drug interactions between six widely used cardiovascular-protective Chinese herbs and ARV drugs. The herbs were selected based on their traditional usage and relevance to cardiovascular support in Chinese herbal medicine practice. The focus of the present study was on the modulation of CYP enzymes—the major pathway for ARV drugs metabolism and a common site for clinically significant drug-drug interactions. By synthesising the available evidence on CYP enzyme activity, this review aimed to identify preliminary safety concerns and clarify the potential risks of pharmacokinetic interference.

### 1.1 Role of metabolising enzyme in drug-drug interactions

Metabolizing enzymes play a critical role in drug pharmacokinetics and dynamics by facilitating the biotransformation, breakdown, and elimination of drugs from the body. These enzymes, primarily expressed in hepatocytes, include CYPs, uridine 5′-diphospho-glucuronosyltransferases (UGTs), and glutathione S-transferases (GSTs) (Peters et al., 1989). CYP3A4 is especially important, metabolizing approximately 50% of all marketed drugs, including many ARV medications. Other CYPs involved in ARV drugs metabolism are CYP2B6, CYP2C9, CYP2C19, and CYP2D6 (see Supplementary Table 1). Although UGTs catalyse phase II metabolism and GSTs contribute to detoxification, our systematic review focussed on CYPs, as they mediate phase I metabolism and are responsible for most clinically significant drug-drug interactions associated with ARV therapy.

ARV drugs can act as substrates, inhibitors, or inducers of CYPs (Gong et al., 2019). Such interactions can alter drug pharmacokinetics, with inhibition increasing concentration and toxicity risk, and induction lowering levels and efficacy. Because maintaining stable drug exposure is critical for viral suppression and resistance prevention, understanding CYP modulation is essential for evaluating herb–drug interaction risks when Chinese herbal medicines are used alongside ARV therapy.

### 1.2 Role of nuclear receptors in drug-drug interactions

The Pregnane X Receptor (PXR) and Constitutive Androstane Receptor (CAR) are key nuclear receptors involved in regulating the expression of drug-metabolizing enzymes, particularly CYPs (Wang Y. M et al., 2012). PXR, which plays a more prominent role in interactions involving ARV drugs, primarily induces CYP3A4, enhancing drug metabolism and clearance. CAR regulates enzymes including CYP2B6 and phase II enzymes that contributing to detoxification pathways.

### 1.3 *In vitro* assays for studying drug-drug interactions

*In vitro* modulation assays are typically classified based on their mechanistic focus: inhibition or induction of enzyme activity, mRNA expression, or protein expression. Enzyme inhibition is commonly assessed using human liver microsomes (HLMs), which are enriched with phase I drug-metabolizing enzymes, particularly CYP isoforms. These assays typically quantify the metabolism of selective CYP substrates using liquid chromatography–tandem mass spectrometry (LC-MS/MS) (Chen et al., 2023; Lv et al., 2015). This model is particularly suitable for evaluating the inhibitory potential of herbal compounds, offering high sensitivity and specificity to detect direct, reversible, and mechanism-based inhibition, without the confounding influence of cellular regulatory pathways.

In contrast, enzyme induction studies require intact transcriptional machinery, which is preserved in hepatocyte-derived cell lines or primary human hepatocytes. Induction is driven by nuclear receptor-mediated activation (e.g., via PXR, CAR), which leads to upregulation of CYP mRNA and protein synthesis. Induction assays often utilize qPCR, Western blotting, enzyme activity assays, and nuclear receptor activation assays to quantify changes in enzyme expression and activity.

Together, these *in vitro* assays are crucial for evaluating drug–drug or herb-drug interactions by assessing how substances modulate metabolizing enzymes. Inhibition assays measure parameters such as IC50, Ki, competitive binding, and time-dependent inhibition to characterize enzyme suppression. Induction assays, on the other hand, typically measure fold changes in mRNA and protein expression levels of drug-metabolising enzymes—most commonly CYP isoforms—in liver cells (Wang et al., 2011; Wang Y. G et al., 2012). These outcome measures are commonly used to predict whether a compound may modulate enzyme activity in the context of potential herb–drug interactions.

### 1.4 Selection of commonly used cardiovascular herbs

Chinese herbal medicine has been used for millennia in the management of CVD. Building on our previous systematic review (Li et al., 2022), we focused on six commonly used herbs with cardiovascular, anti-inflammatory, and immune-supportive properties: Dan Shen (*Salvia miltiorrhiza*), Huang Qi (*Astragalus membranaceus/propinquus*), Bai Zhu (*Atractylodes macrocephala*), Chuan Xiong (*Ligusticum chuanxiong*), Dang Gui (*Angelica sinensis/polymorpha*), and Gan Cao (*Glycyrrhiza uralensis/glabra*) (Chao and Lin, 2011; Jung et al., 2020; Ran et al., 2011; Su et al., 2021; Wahab et al., 2021; Yang et al., 2021). These herbs were selected for their potential to be combined into a formula addressing key CVD risk factors, such as hs-CRP, IL-6, and TNF, relevant to this population.

In Chinese herbal medicine, a single herb may encompass multiple botanical varieties with distinct Latin names (e.g., *G. uralensis* vs. *Glycyrrhiza glabra*). To ensure consistency and avoid confusion, we have standardized herb nomenclature using Pinyin throughout the manuscript.

Dan Shen invigorates blood and dispels stasis; its phenolic acids and tanshinones exert anti-inflammatory and cardioprotective effects, improve lipid profiles, and inhibit platelet activation (Liu et al., 2016; Stumpf et al., 2013). Huang Qi tonifies Qi and strengthens the Spleen; its astragalosides, polysaccharides, and flavonoids protect vascular endothelium via anti-inflammatory and growth factor–mediated pathways (Su et al., 2021; Wang et al., 2016). Bai Zhu strengthens the Spleen, dries dampness, and supports gut health; its polysaccharides, volatile oils, and lactones modulate intestinal microecology and attenuate vascular inflammation (Yang et al., 2021).

Chuan Xiong moves Qi and blood to relieve pain; its ligustilide, ferulic acid, and chuanxiongzine improve lipid metabolism, inhibit platelet aggregation, and suppress inflammatory cytokines (Ran et al., 2011). Dang Gui nourishes and invigorates blood; its ferulic acid, ligustilide, and phthalides reduce inflammation, regulate lipids, and may improve insulin sensitivity (Chao and Lin, 2011; Guo et al., 2009). Gan Cao harmonises formulas and mitigates toxicity; its glycyrrhizin and flavonoids exert anti-inflammatory effects through NF-κB and TNF inhibition (Wahab et al., 2021).

### 1.5 Objectives

This review systematically evaluates *in vitro* evidence on herb-drug interactions involving six cardioprotective herbs and ARV drugs, with a particular focus on the modulation of CYP enzymes. The primary aim is to identify potential safety concerns related to pharmacokinetic interactions and provide preliminary insights that can inform future clinical and translational research.

## 2 Methods

### 2.1 Eligibility criteria

#### 2.1.1 Inclusion criteria

Only peer-reviewed journal articles written in English or Chinese were included. To capture a broad range of results, we used popular components in the search terms. However, the screening process required narrowing the selection to ensure consistency and relevance. The reference components were based on the Chinese Pharmacopoeia (Chinese Pharmacopoeia Commission, 2020), as shown in Table 1. We included studies involving extracts or reference components from Dan Shen, Huang Qi, Bai Zhu, Dang Gui, Chuan Xiong or Gan Cao.

**TABLE 1:** Chemical profiles of reference components in selected herbal products.

Herbal Product	References Components	Chemical Formula
*Dan Shen*	Tanshinone IIA	C_19_H_18_O_3_
	Tanshinone I	C_18_H_12_O_3_
	Cryptotanshinone	C_19_H_20_O_3_
	Salvianolic Acid B	C_36_H_30_O_16_
*Huang Qi*	Astragaloside IV	C_41_H_68_O_14_
	Calycosin-7-O-beta-D-glucoside	C_22_H_22_O_10_
*Bai Zhu*	Atractylone	C_15_H_20_O
*Dang Gui*	Ferulic Acid	C_10_H_10_O_4_
*Chuan Xiong*	Ferulic Acid	C_10_H_10_O_4_
*Gan Cao*	Glycyrrhizic Acid	C_42_H_62_O_16_
	Liquiritin	C_21_H_22_O_9_

Reference components are the specific chemical compounds listed in the pharmacopeia as standard markers used to verify the quality, authenticity, and consistency of an herbal preparation. These components serve as benchmarks to ensure that the preparation meets established criteria for potency, purity, and composition.

#### 2.1.2 Exclusion criteria

Studies that did not use the six herbal extracts or their reference components, or studies without outcomes related to ARV drugs metabolizing enzymes, were not eligible. Additional exclusions included studies using recombinant CYPs, non-human liver microsomes or cell lines, and those with poor methodological quality.

We excluded studies using recombinant enzymes or non-human cell models to ensure included data reflected human-relevant hepatic metabolism, gene regulation, and enzyme induction/inhibition dynamics necessary for clinically meaningful herb–drug interaction assessment.

#### 2.1.3 Grouping of studies by assay type

Studies were grouped by assay methodology based on whether they measured induction or inhibition. The evaluation of induction and inhibition requires different assays due to their distinct biological mechanisms. Induction involves increasing enzyme expression through gene transcription, often mediated by nuclear receptors like PXR or CAR, necessitating the use of living cell systems to replicate these complex processes. In contrast, inhibition directly reduces enzyme activity via interactions with the enzyme’s active site, which can be measured using simplified systems like human liver microsomes, as these contain pre-formed enzymes suitable for assessing enzymatic activity. This grouping enabled systematic bias comparisons and provided a structured framework for analysing reported outcomes in this systematic review.

### 2.2 Information sources and search strategy

The English databases searched included MEDLINE (via PubMed) and Embase (Excerpta Medica database), focusing on health-related literature, while Scopus was utilized as a multidisciplinary resource. Additionally, the Chinese National Knowledge Infrastructure (CNKI) database was searched to capture relevant studies published in Chinese.

It is important to note that the Cochrane database was not included in this search, as it primarily focuses on clinical trials and observation studies. Searches of all sources were last conducted on 31 May 2024, incorporating literature from the earliest available year to the search date, ensuring the inclusion of the most current and relevant studies.

### 2.3 Data collection and synthesis

#### 2.3.1 Data extraction

The search aimed to identify studies on the modulation of CYP enzymes relevant to ARV drugs metabolism or the activation of nuclear receptors by the six herbs. Key targets included CYP3A4, CYP2B6, CYP2C9, CYP2C19, CYP2D6, and nuclear receptors PXR and CAR to assess potential herb-drug interactions with ARV drugs. Search results are detailed in Supplementary Tables 2–5.

Records from all databases were combined, and duplicates were removed. Titles and abstracts were screened to exclude irrelevant studies, and those focussed solely on clinical trials or *in vivo* data. Reviews, opinion pieces, theses, and conference abstracts were also excluded, with full-text screening conducted for the remaining records. Data management was performed using Excel spreadsheet and EndNote TM 2.0. Excluded studies are tracked in Supplementary Table 6. The selection outcome was verified by one independent reviewer. The study selection process was conducted following the PRISMA guidelines (Moher et al., 2009), including identification, screening, eligibility assessment, and final inclusion of studies.

#### 2.3.2 Data categorization

Data collection followed the SciRAP assessment tool guidelines, with two reviewers involved—one collecting and one verifying the data. Given this review’s focus on identifying modulation trends rather than outcome magnitudes, obtaining missing data figures was not prioritized, and no automation tools were used. The collected data were entered into a spreadsheet for analysis, recording outcome measurements and highlighting statistically significant findings. The data were then classified into three categories: induction, inhibition, or no effect.

#### 2.3.3 Data analysis

Due to substantial variability in experimental designs, extract preparations, outcome measures, and different assay protocols across studies, formal meta-analysis or statistical pooling was not feasible or meaningful. Instead, we summarized results in tables and histograms to facilitate comparison. We also performed a descriptive subgroup analysis focused on aqueous extracts given their clinical relevance.

### 2.4 Risk of bias assessment

To assess the risk of bias in the included studies, we utilized the Science in Risk Assessment and Policy (SciRAP) *in vitro* tool (version 2.1, 2024), developed by the Department of Environmental Science at Stockholm University and the Institute of Environmental Medicine at Karolinska Institute.

SciRAP is a framework that provides structured tools for evaluating the reliability and relevance of toxicological and ecotoxicological studies in chemical risk assessment. This tool evaluates study reliability based on 24 criteria for “reporting quality” and 16 criteria for “methodological quality”.

Prior to the assessment, we consulted with the program designer and held thorough discussions within our team regarding the data coding process and the relevance of specific questions from the SciRAP checklist. We excluded four reporting quality questions (#3, 8, 9, and 18) and three methodological quality questions (#1, 2, and 13).

The excluded checklist items were deemed less applicable to the studies included in this review. For example, reporting checklist item #3 and methodological items #1 and #2 address solubility and purity of individual herbal constituents, which is generally not feasible given the complexity of herbal extracts and the variability introduced by different extraction methods.

Similarly, reporting checklist item #8 relates to validation of metabolic competence; however, the use of human cell lines for induction studies and human liver microsomes for inhibition studies is well-established and widely recognized as appropriate for these purposes. While liver microsomes cannot capture the full metabolic profile due to the absence of certain cytosolic enzymes and cellular architecture, supplementation with cofactors such as NADPH allows reliable assessment of specific enzyme activities, making them a standard system for evaluating drug–enzyme interactions. As such, it is less common for studies employing these established systems to conduct and explicitly report separate assessments of metabolic competence.

Reporting checklist item #9 was also considered less relevant, as it pertains to proliferating cell cultures and is not applicable to non-proliferating microsomal preparations. Finally, reporting checklist item #18 and methodological item #13, which address detailed time-course analyses, were not appropriate in this context, since most studies measured outcomes at a single time point in line with common practice for enzyme inhibition and induction assays.

Strict application of these items would have unfairly penalized studies for methodological choices that reflect accepted standards or involve criteria that are not feasible or relevant, rather than true reporting deficiencies.

Contextual remarks were noted for each checklist question to align with revised assessment guidelines, and all evaluations were cross-checked by a second reviewer to ensure consistency and accuracy. The assessment of study relevance was not conducted in this review because the identity of the tested substances was strictly restricted to meet the selection criteria. Additionally, the test systems and study endpoints were intentionally broadened to include various test systems, concentrations used, and endpoints studied. No automation tools were employed.

## 3 Results

### 3.1 Study selection

**FIGURE 1:**
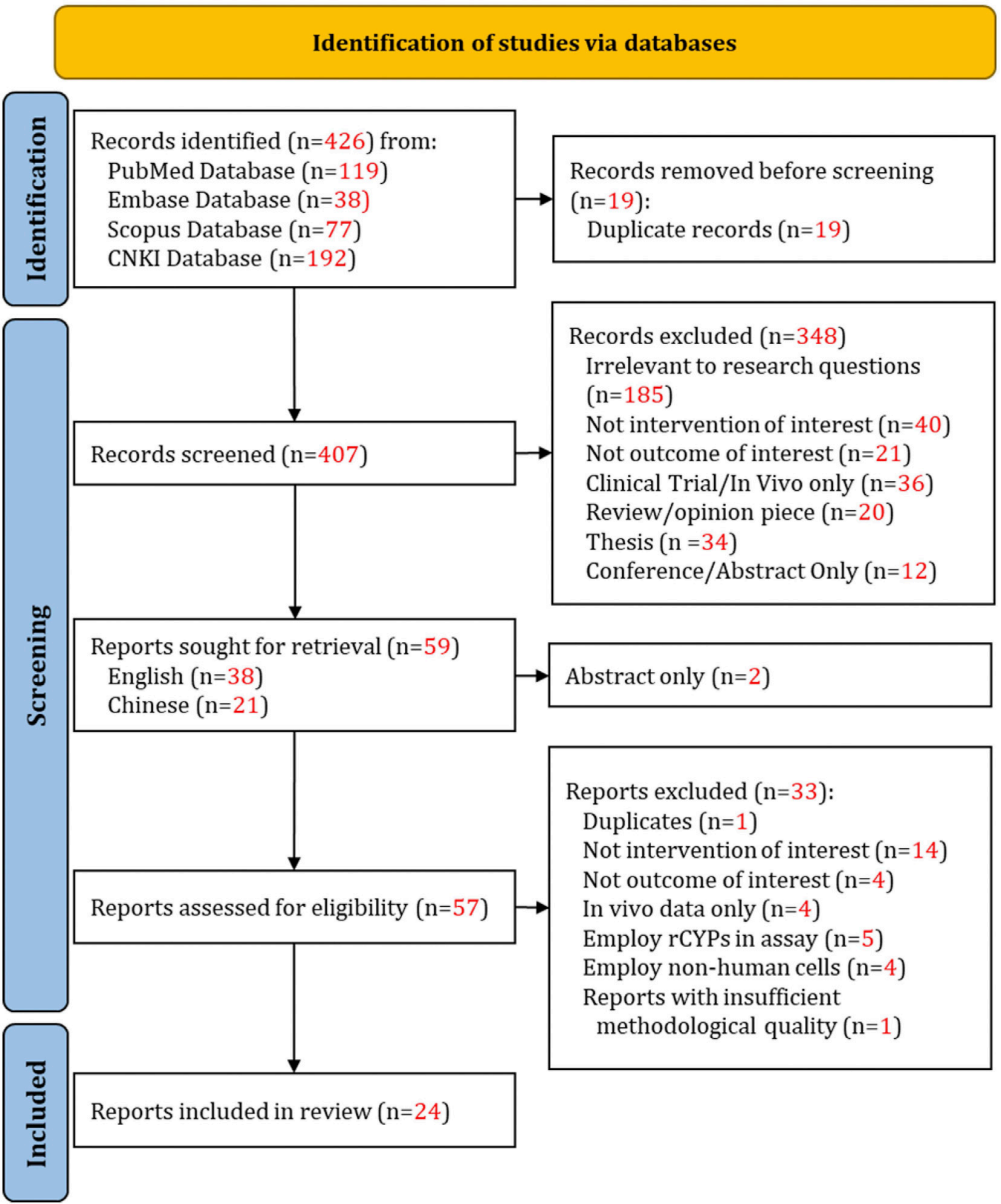
PRISMA flow diagram of the literature search, screening, and selection process for eligible studies.

Figure 1 illustrates the study identification, screening, and inclusion process, with the numerical outcomes of articles at each stage, in accordance with PRISMA guidelines. A total of 59 full-text articles were retrieved and assessed. After matching the articles against the selection criteria, 24 studies were deemed eligible for this review.

### 3.2 Characteristics of studies included in this systematic review

The basic characteristics of the studies are summarised in Table 2. Dan Shen and Gan Cao were the most examined herbs, appearing in ten and nine investigations, respectively. Huang Qi was reported in five studies, Dang Gui in three, and both Chuan Xiong and Bai Zhu appeared in one study each.

**TABLE 2:** Summary of study characteristics, extract types, components tested, methodologies, and outcomes by herb.

(Author/year)	Herb	Plant variety	Extract type	Compounds	Materials	Markers	Methodologies	Outcomes
Dong et al. (2008)	Bai Zhu	*A. macrocephala*	Aq		HepG2	PXR	LR	PXR Act
Li et al. (2020)	Chuan Xiong	L. chuanxiong	Aq	Ferulic Acid	Caco-2	3A4	WB, ELISA	ProtExpr, EnzAct
Huang et al. (2011)	Dan Shen	*S. miltiorrhiza*		Cryptotanshinone, Salvianolic acid B, Tanshinone I, Tanshinone IIA	LS174T	CAR	LR	CAR Act
Ma and Zhang (2018)		*S. miltiorrhiza*		Cryptotanshinone, Tanshinone IIA	HepG2	3A4	WB	ProtExpr
Mao et al. (2012)		*S. miltiorrhiza*	EtOH		HLM	3A4	LC-MS/MS	RelAct, IC50
Wang and Yeung (2012)		*S. miltiorrhiza*	EtOH		HLM	3A4	HPLC	IC50, InhibType
Wang et al. (2010)		*S. miltiorrhiza*		Cryptotanshinone, Tanshinone I, Tanshinone IIA	HLM	2C9, 3A4	HPLC	IC50, Ki, InhibType
Wang et al. (2011)		*S. miltiorrhiza*		Salvianolic acid B	HepG2	3A4	RT-PCR	mRNAExpr
Wang et al. (2015)		*S. miltiorrhiza*		Cryptotanshinone	Chang Liver	2C9, 3A4	RT-PCR, WB	mRNAExpr, ProtExpr
Xu et al. (2015)		*S. miltiorrhiza*		Cryptotanshinone, Tanshinone I, Tanshinone IIA, Salvianolic acid B	HLM	2C9, 2C19, 2D6, 3A4	LC-MS/MS	InhibRate, IC50
Yu et al. (2009)		*S. miltiorrhiza*	Aq, EtOH	Cryptotanshinone, Tanshinone I, Tanshinone IIA	HepG2, LS174T	PXR, 3A4	LR, RT-PCR	PXR Act, mRNAExpr
Hu et al. (2015)		*S. miltiorrhiza*	Aq, EtOH	Cryptotanshinone, Tanshinone I, Tanshinone IIA	HLM	2C19	HPLC	IC50, Ki, InhibType
Li et al. (2020)	Dang Gui	*A. sinensis*	Aq	Ferulic Acid	Caco-2	3A4	WB, ELISA	ProtExpr, EnzAct
Mao et al. (2012)		A. sinensis	EtOH		HLM	3A4	LC-MS/MS	RelAct, IC50
Sevior et al. (2010)		A. polymorpha	Aq, MeOH		HLM	2B6, 2C9, 2C19, 2D6, 3A4	LC/MS-MS	InhibRate, IC50
Chen et al. (2023)	Gan Cao	*G. uralensis*	EtOH		HLM	2B6, 2C9, 2C19, 2D6, 3A4	UHPLC-MS/MS	RemAct
Guo et al. (2014)		*G. uralensis*	Aq		HLM	3A4	LC-MS/MS	RelAct, IC50
He et al. (2009)		*G. uralensis*		Liquiritin	HLM	2C9, 2C19, 2D6, 3A4	LC-MS/MS	IC50
Li et al. (2023)		*G. uralensis*		Liquiritin	HepG2	2C9, 3A4	RT-PCR, WB	mRNAExpr, ProtExpr
Lv et al. (2015)		*G. uralensis*		Glycyrrhetinic acid	HLM	2C9, 2C19, 2D6, 3A4	HPLC-MS/MS	IC50, Ki, InhibType
Mao et al. (2012)		G. uralensis	EtOH		HLM	3A4	LC-MS/MS	RelAct, IC50
Sevior et al. (2010)		*G. glabra*	Aq, MeOH		HLM	2B6, 2C9, 2C19, 2D6, 3A4	LC/MS-MS	InhibRate, IC50
Wang Y. G. et al. (2012)		*G. uralensis*		Glycyrrhizin	HepG2	PXR, 3A4	LR, RT-PCR, WB	PXR Act, mRNAExpr, ProtExpr
Zhang et al. (2022)		*G. uralensis*		Glycyrrhizin, Liquiritin	L02	PXR	LR	PXR Act
Lau et al. (2013)	Huang Qi	*A. propinquus*	EtOH		HLM, LS180	3A4, PXR	FA, LR	IC50, PXR Act
Mao et al. (2012)		A. membranaceus	EtOH		HLM	3A4	LC-MS/MS	RelAct, IC50
Zhang et al. (2017)		*A. membranaceus*		Astragaloside IV	HLM	2C9	HPLC	InhibRate
Xia et al. (2012)		*A. membranaceus*		Astragaloside IV, Liquiritin	LS174T	PXR	LR	PXR Act
Zhang et al. (2013)		*A. membranaceus*	Inj, Gran		LS174T	PXR, CAR	LR	PXR Act, CAR Act

Abbreviations: Extract Type–Aq, Aqueous; EtOH, Ethanolic; MeOH, Methanolic; Inj, Injection; Gran, Granule.

Materials - HLM, human liver microsomes; HepG2, human hepatocellular carcinoma cell line; LS174T, human colon adenocarcinoma cell line; LS180, Human Colon Adenocarcinoma Cell Line; Caco-2, human colorectal adenocarcinoma cell line; L02, Cervical cancer line HeLa (previously misidentified as human foetal hepatocyte cell line) (Weiskirchen, 2023); Chang Liver, Human Liver Epithelial Cell Line.

Markers - 2B6, Cytochrome P450 Family 2 Subfamily B Member 6, and so on in a similar manner; PXR, Pregnane X Receptor; CAR, constitutive androstane receptor.

Methodologies - UHPLC-MS/MS, Ultra-High-Performance Liquid Chromatography Tandem Mass Spectrometry; LC-MS/MS, liquid chromatography tandem mass spectrometry; HPLC, High-Performance Liquid Chromatography; HPLC-MS/MS, High-Performance Liquid Chromatography Tandem Mass Spectrometry; ELISA, Enzyme-Linked Immunosorbent Assay; FA, fluorometric assay; LR, luciferase reporter; RT-PCR, reverse transcription polymerase chain reaction; WB, western blot.

Outcomes–RemAct, Remaining Activity; RelAct, Relative Activity; IC50, Half-Maximal Inhibitory Concentration; Ki, Inhibition Constant; InhibType, Inhibition Type; InhibRate, Inhibition Rate; EnzAct, Enzyme Activity; ProtExpr, Protein Expression; mRNAExpr, mRNA, expression; PXR Act, Pregnane X Receptor Activation; CAR Act, Constitutive Androstane Receptor Activation.

Outcome Measures and Definitions: Remaining Activity: The fraction of the enzyme’s original activity that persists after the application of an inhibitor. Relative Activity: The comparison of enzyme activity in the presence *versus* the absence of an inhibitor. Ki: Inhibition Constant is related to the affinity of the inhibitor and is the concentration required to produce half maximal inhibition of enzyme activity. IC50: Half-Maximal Inhibitory Concentration, the concentration of an inhibitor at which the activity of an enzyme or receptor is reduced by 50% under the conditions of the assay.

Among the purified compounds, Cryptotanshinone was examined in seven studies, while Tanshinone IIA appeared in six. Tanshinone I and Liquiritin were each investigated in five and four studies, respectively. Salvianolic Acid B was included in three studies, while Glycyrrhizin and Astragaloside IV were each reported in two studies. Other compounds, such as Ferulic Acid and Glycyrrhetinic Acid, appeared in only one study each.

Across the 24 studies reviewed, ethanolic and aqueous extracts were equally represented, each being used in six studies. Methanolic extracts, as well as injection or granule preparations, appeared in just one study each.

Inhibition assays were conducted in twelve studies to assess herbal effects on CYP enzymes. For induction assays, the HepG2 cell line was most used, appearing in six studies, followed by the LS174T cell line in four studies. Other cell lines, including L02, Chang Liver, Caco-2, and LS180, were each used in one study.

CYP3A4 was the most frequently studied target enzyme, followed by CYP2C9, CYP2C19, and CYP2D6, with CYP2B6 less commonly explored. The PXR, a key regulator of CYP expression, was included in seven studies to assess PXR-CYP3A4-mediated herb-drug interactions, while CAR activation was reported in two studies.

Chromatographic and mass spectrometric techniques were commonly applied in inhibition assays, with IC50 values frequently reported, followed by Ki values and types of inhibition. For induction assays, luciferase reporter assays assessed PXR-dependent CYP3A4 induction, while RT-PCR and Western blotting analysed gene expression and protein levels. Fluorometric assays, ELISA, and enzyme activity assessments were less commonly used.

### 3.3 Risk of bias analysis

The adapted SciRAP checklist items and detailed scoring results for each included study are provided in Supplementary Table 7 to ensure transparency and reproducibility of the risk-of-bias assessment.

Table 3 summarizes the reporting and methodological quality of the 24 eligible studies. All studies scored 75% or above, indicating a high standard of quality and reinforcing the reliability of their findings. To enhance the robustness of our analysis, lower-quality studies such as those that did not specify the cell line used or the culturing conditions were excluded, ensuring that the remaining studies adhered to rigorous documentation practices for reproducibility.

**TABLE 3:** Reporting and methodological quality of studies assessed by SciRAP risk of bias tool.

(Author/Year)	Reporting Quality (%)	Methodological Quality (%)
Chen et al. (2023)	97.4	79.2
Dong et al. (2008)	89.5	100
Guo et al. (2014)	89.5	83.3
He et al. (2009)	86.8	100
Hu et al. (2015)	94.7	91.7
Huang et al. (2011)	86.8	100
Lau et al. (2013)	86.8	100
Li et al. (2020)	76.3	92.3
Li et al. (2023)	86.8	100
Lv et al. (2015)	86.8	95.8
Ma and Zhang (2018)	78.9	83.3
Mao et al. (2012)	84.2	83.3
Sevior et al. (2010)	86.8	75
Wang and Yeung (2012)	84.2	87.5
Wang et al. (2010)	81.6	83.3
Wang et al. (2011)	97.4	100
Wang Y. G. et al. (2012)	78.9	83.3
Wang et al. (2015)	89.5	91.7
Xia et al. (2012)	78.9	100
Xu et al. (2015)	81.6	83.3
Yu et al. (2009)	86.8	100
Zhang et al. (2013)	89.5	100
Zhang et al. (2017)	78.9	75
Zhang et al. (2022)	92.1	100

The SciRAP, *in vitro* tool (version 2.1, 2024) was used to assess the risk of bias in the included studies. Developed by Stockholm University and the Karolinska Institute, this tool evaluates study reliability based on 24 criteria for reporting quality and 16 criteria for methodological quality. The reporting quality had an average score of 86.3%, ranging from 76.3% to 97.4%, with a mode of 86.8%. The methodological quality had an average score of 90.8%, ranging from 75.0% to 100.0%, with a mode of 100.0%.

Only 24 studies remained eligible after a stringent selection process, underscoring the importance of each included study in contributing valuable insights into the effects of selected herbs on metabolizing enzymes and drug-drug interactions. Although some outcomes were not the primary focus and detailed statistical data were occasionally lacking, the figures and narrative descriptions of modulation effects contribute meaningfully to our analysis.

### 3.4 Comparative overview of induction and inhibition assays

**FIGURE 2:**
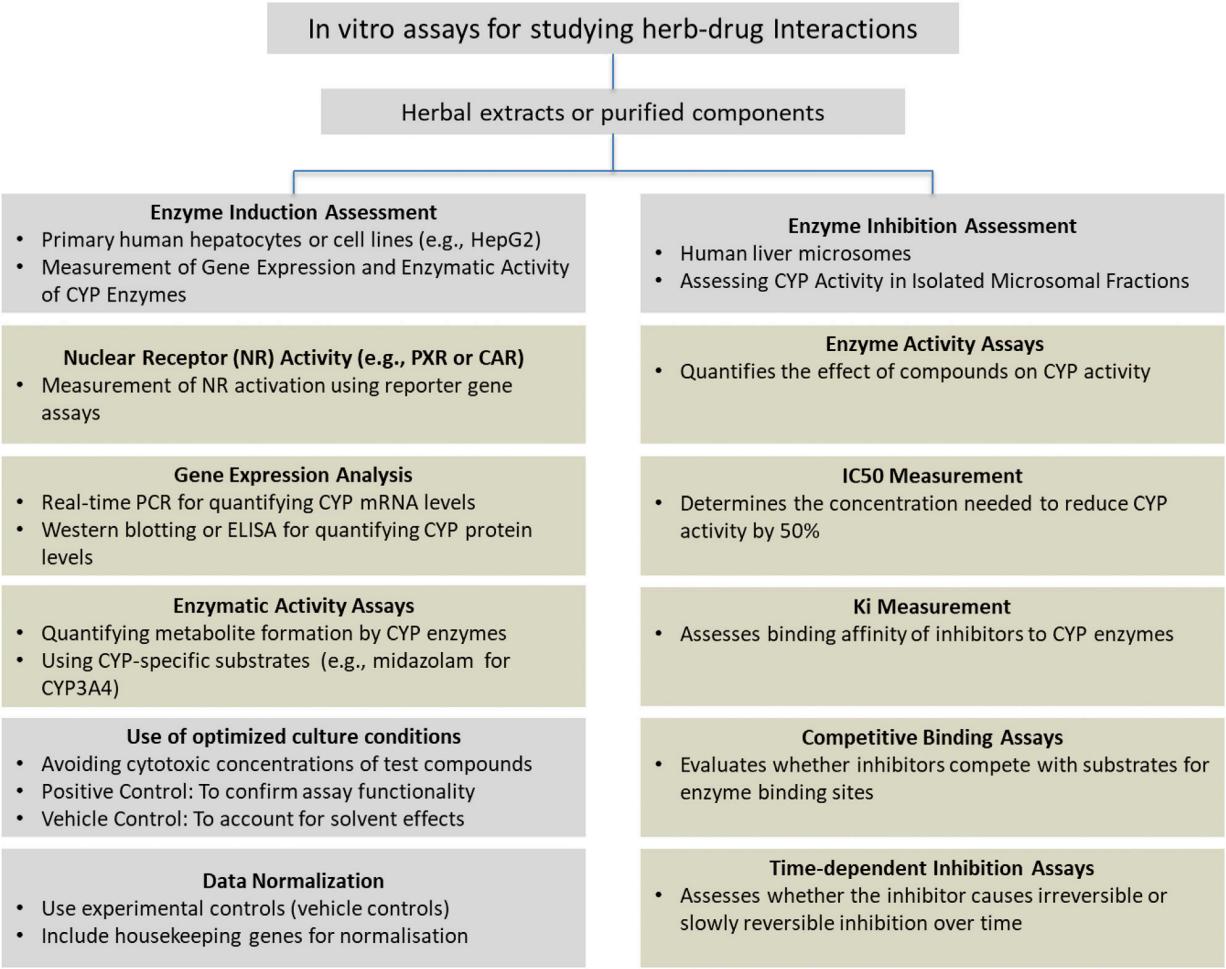
Experimental methodology for investigating herb-drug interactions via *in vitro* assays.

A summary of the methodology used to investigate herb-drug interactions is presented in Figure 2. This figure outlines the key experimental approaches used for assessing the induction or inhibition of CYP enzymes.

Tables 4 further summarize the experimental protocols for induction and inhibition assays, detailing key parameters such as replicates, extract concentrations, procedures, and positive controls.

**TABLE 4:** Summary of experimental replicates, concentrations, procedures, and controls used in induction and inhibition assays.

Assay Type	Author/Year	Replicates	Concentrations	Experimental Procedures	Positive Controls
Induction	Dong et al. (2008)	6	200 mg/L	HepG2 cells transfected with 0.9 µg plasmid +1.8 µg Lipofectamine™, 6 h; treated 24 h	Rifampin
	Huang et al. (2011)	3	2.5–40 µM	70%–80% confluent cells; transfected with CAR plasmids 6 h; treated 24 h	CITCO
	Lau et al. (2013)	4	33–250 μg/mL	LS180 cells; transfected with plasmids using Nanofectin™ 24 h; treated 24 h	Ketoconazole
	Li et al. (2020)	3	64–640 mg/mL	Caco-2 cells, treated 12 h at log phase	Ketoconazole
	Li et al. (2023)	3	20 μM	HepG2 cells starved 12 h; treated 48 h	Rifampin, Carbamazepine
	Ma and Zhang (2018)	3	5 ng/mL	HepG2 cells treated 48 h	Cryplotanshine
	Wang et al. (2011)	6	1, 10 μM	HepG2 cells seeded 18 h; treated 24 h	Rifampin
	Wang Y. G. et al. (2012)	3	50–500 μM	HepG2 cells 80% confluent; treated serum-free medium; mRNA 12 h, WB 24 h	Rifampin
	Wang et al. (2015)	NA	10, 100 μM	Chang Liver cells; treated 1, 3, 7 days	Rifampin
	Xia et al. (2012)	5	5–80 μM	LS174T cells; 6 h transfection; treated 24 h	Rifampin
	Yu et al. (2009)	3	Extract: 100 μg/mL; Compound: 2 μM	PXR activation: HepG2 36 h post 6 h transfection; LS174T mRNA 3 days	Rifampin
	Zhang et al. (2013)	3	AI: 62.5–1,000 mg/mL; AG: 62.5–1,000 μg/mL	LS174T cells treated 24 h post 4 h transfection	Rifampin
	Zhang et al. (2022)	3	10, 20, 50 μM	L02 cells treated 24 h at 70%–80% confluence	Rifampin
Inhibition	Chen et al. (2023)	2	5 μg/mL	HLM (0.2 mg/mL) + extract +1.3 mM NADPH + substrate, 37 °C, 10 min	NA
	Guo et al. (2014)	3	0–10 mg/mL	HLM (0.9 mg/mL) pre-incubated 15 min, then probe 30 min	Ketoconazole
	He et al. (2009)	3	2.5–100 μM	HLM (0.25 mg/mL) pre-incubated with substrate 5 min, NADPH 20 min	Loratadine
	Hu et al. (2015)	3	6.25–100 μM	HLM (0.8 mg/mL) incubated with tanshinones 5 min, NADPH 1 h	Nootkatone
	Lau et al. (2013)	2	Serial μg/mL	Herbal extract pre-incubated 15 min; then Supersomes™ 30 min	Ketoconazole
	Lv et al. (2015)	3	0.01–100 μM	HLM pre-incubated with NADPH 0,10,30 min; midazolam 37 °C 30 min	Midazolam
	Mao et al. (2012)	3	0.2–200 μg/mL	HLM (0.3 mg/mL) pre-incubated 15 min; testosterone +1 mM NADPH 30 min	Ketoconazole
	Sevior et al. (2010)	2	20–500 μg/mL	Microsome (0.5 mg/mL) pre-incubated 2 min; NADPH 20 min	NA
	Wang and Yeung (2012)	3	2.5–20 μg/mL	HLM (0.8 mg/mL) pre-incubate with NADPH 10 min, 800 rpm, 37 °C	Ketoconazole
	Wang et al. (2010)	NA	2C9: 2.5–40 μM; 3A4: 0.25–25 μM	HLM (0.8 mg/mL) pre-incubated 5 min; NADPH 1 h	Sulfaphenazole, Ketoconazole
	Xu et al. (2015)	2	10 μM	HLM (0.5 mg/mL) pre-incubate 5 min; then NADPH for 2C9/2C19 60 min, 2D6 30 min, 3A4 4 min	NA
	Zhang et al. (2017)	2	3.91–250 μM	Microsomal protein 0.5 g/L pre-incubated 5 min; NADPH 60 min	NA

Induction assays typically involved transfecting reporter plasmids into cell lines, followed by 12–72 h of incubation with herbal products to measure mRNA, protein expression, or luciferase activity.

Inhibition assays used shorter incubation periods, with herbal products preincubated with the substrate, followed by enzyme activation with a coenzyme. The reaction was stopped at a specific time to measure enzyme activity by assessing metabolite production, ensuring consistency and minimizing variability.

### 3.5 Studies outcome and result synthesis

**FIGURE 3:**
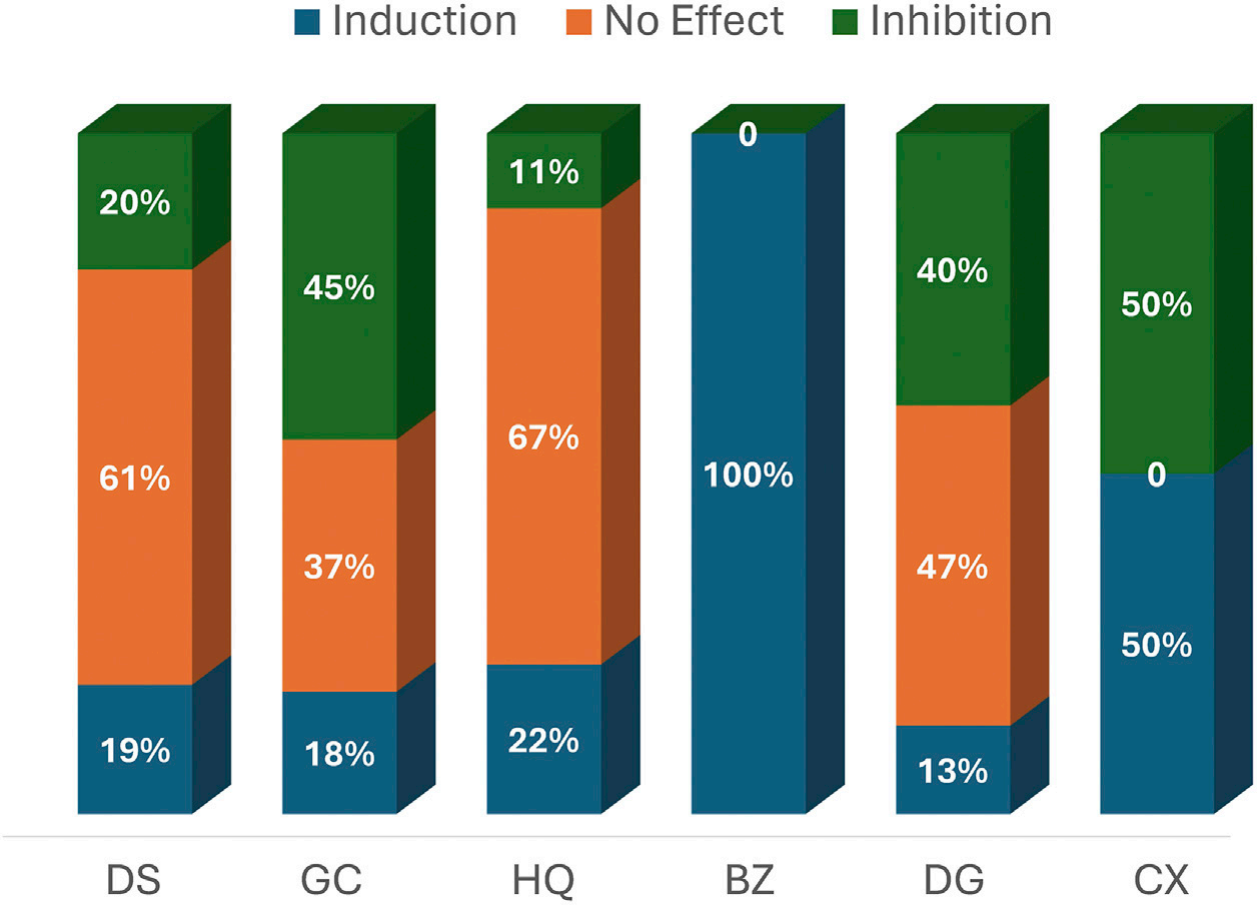
Percentage Distribution of Reported CYP Modulation Effects by Herb. Abbreviations: DS, Dan Shen (*Salvia miltiorrhiza*); GC, Gan Cao (*Glycyrrhizae uralensis/glabra*); HQ, Huang Qi (*Astragali membranaceus/propinquus*); BZ, Bai Zhu (*Atractylodes macrocephala*); DG, Dang Gui (*Angelica sinensis/polymorpha*); CX, Chuan Xiong (*Ligusticum chuanxiong*).

Table 5 provides detailed information from the induction and inhibition assays, while Table 6 presents a concise summary of their modulation outcomes. Across all the herbs examined, combining results from various forms of intervention—aqueous or organic extracts, or reference compounds—“no effect” was the most frequently reported outcome. Dan Shen demonstrated no significant effect on enzyme modulation in 30 assays (61%), making it the herb with the highest number of neutral outcomes. Gan Cao followed with 14 assays (37%) reporting no effect, while Dang Gui and Huang Qi showed neutral outcomes in 7 (47%) and 6 (67%) assays, respectively, Figure 3.

**TABLE 5:** Modulatory effects of selected herbal extracts and relevant bioactive compounds on cytochrome P450 (CYP) enzymes: Inhibition (INH), No effect (NE), or induction (IND).

Dan Shen							
Author/Year	Extract/Compound	CYP	Marker/Test	Outcome	INH	NE	IND
Wang/2010	C-CT	2C9	IC_50_	23.86 μM	↓		
Xu/2015	C-CT	2C9	IR%	<10		•	
Wang/2015	C-CT	2C9	mRNA-FC	0.81–1.14		•	
Wang/2015	C-CT	2C9	Protein-FC	0.83–1.19		•	
Xu/2015	C-SAB	2C9	IR%	<10		•	
Wang/2010	C-TI	2C9	IC_50_	>100 μM		•	
Xu/2015	C-TI	2C9	IR%	<10		•	
Wang/2010	C-TIIA	2C9	IC_50_	>100 μM		•	
Xu/2015	C-TIIA	2C9	IR%	<10		•	
Hu/2015	C-CT	2C19	IC_50_	>100 μM		•	
Xu/2015	C-CT	2C19	IR%	<10		•	
Xu/2015	C-SAB	2C19	IR%	<10		•	
Hu/2015	C-TI	2C19	IC_50_	>100 μM		•	
Xu/2015	C-TI	2C19	IR%	<10		•	
Hu/2015	C-TIIA	2C19	IC_50_	>100 μM		•	
Xu/2015	C-TIIA	2C19	IR%	<10		•	
Hu/2015	E-EtOH	2C19	IC_50_	30.9 μg/mL	↓		
Hu/2015	E-H2O	2C19	IC_50_	>100 μM		•	
Xu/2015	C-CT	2D6	IR%	<10		•	
Xu/2015	C-SAB	2D6	IR%	<10		•	
Xu/2015	C-TI	2D6	IR%	<10		•	
Xu/2015	C-TIIA	2D6	IC_50_	13.47 μM	↓		
Xu/2015	C-TIIA	2D6	IR%	>10	↓		
Huang/2011	C-CT	3A4	CAR-3A4-FC	NE		•	
Wang/2010	C-CT	3A4	IC_50_	>100 μM		•	
Xu/2015	C-CT	3A4	IR%	<10		•	
Wang/2015	C-CT	3A4	mRNA-FC	0.80 *	↓		
Yu/2009	C-CT	3A4	mRNA-FC	4.4 ***			↑
Ma/2018	C-CT	3A4	Protein-FC	2.4 *			↑
Wang/2015	C-CT	3A4	Protein-FC	0.91		•	
Yu/2009	C-CT	3A4	PXR-3A4-FC	7.3 ***			↑
Huang/2011	C-SAB	3A4	CAR-3A4-FC	IND^#^			↑
Xu/2015	C-SAB	3A4	IC_50_	12.35 μM	↓		
Xu/2015	C-SAB	3A4	IR%	>10	↓		
Wang/2011	C-SAB	3A4	mRNA-FC	0.7 *	↓		
Huang/2011	C-TI	3A4	CAR-3A4-FC	NE		•	
Wang/2010	C-TI	3A4	IC_50_	>100 μM		•	
Xu/2015	C-TI	3A4	IR%	<10		•	
Yu/2009	C-TI	3A4	mRNA-FC	NE		•	
Huang/2011	C-TIIA	3A4	CAR-3A4-FC	IND^#^			↑
Wang/2010	C-TIIA	3A4	IC_50_	>100 μM		•	
Xu/2015	C-TIIA	3A4	IR%	<10		•	
Yu/2009	C-TIIA	3A4	mRNA-FC	3.1 **			↑
Ma/2018	C-TIIA	3A4	Protein-FC	2.1 *			↑
Yu/2009	C-TIIA	3A4	PXR-3A4-FC	4.3 ***			↑
Wang & Yeung/2012	E-EtOH	3A4	IC_50_	17.5 μg/mL	↓		
Yu/2009	E-EtOH	3A4	PXR-3A4-FC	2.6 **			↑
Mao/2012	E-EtOH	3A4	RA%	<25	↓		
Yu/2009	E-H2O	3A4	PXR-3A4-FC	NE		•	

Gan Cao							
Author/Year	Extract/Compound	CYP	Marker/Test	Outcome	INH	NE	IND
Chen/2023	E-EtOH	2B6	RA-R%	49.1	↓		
Sevior/2010	E-H2O	2B6	IC_50_	>100 μg/mL		•	
Sevior/2010	E-MeOH	2B6	IC_50_	4.2 μg/mL	↓		
Lv/2015	C-GA	2C9	IC_50_	42.89 μM	↓		
He/2009	C-LQ	2C9	IC_50_	>50 μM		•	
Li/2023	C-LQ	2C9	mRNA-FC	1.24 *			↑
Li/2023	C-LQ	2C9	Protein-FC	1.41 *			↑
Chen/2023	E-EtOH	2C9	RA-R%	67.3	↓		
Sevior/2010	E-H2O	2C9	IC_50_	>100 μg/mL		•	
Sevior/2010	E-MeOH	2C9	IC_50_	3.6 μg/mL	↓		
Lv/2015	C-GA	2C19	IC_50_	40.26 μM	↓		
He/2009	C-LQ	2C19	IC_50_	>50 μM		•	
Chen/2023	E-EtOH	2C19	RA-R%	94.7	↓		
Sevior/2010	E-H2O	2C19	IC_50_	>100 μg/mL		•	
Sevior/2010	E-MeOH	2C19	IC_50_	3.7–5.1 μg/mL	↓		
Lv/2015	C-GA	2D6	IC_50_	>100 μM		•	
He/2009	C-LQ	2D6	IC_50_	>50 μM		•	
Chen/2023	E-EtOH	2D6	RA-R%	NE		•	
Sevior/2010	E-H2O	2D6	IC_50_	97.2 μg/mL		•	
Sevior/2010	E-MeOH	2D6	IC_50_	13.7 μg/mL	↓		
Lv/2015	C-GA	3A4	IC_50_	8.195 μM	↓		
Lv/2015	C-GA	3A4	Ki	1.57 μM	↓		
Wang/2012	C-GL	3A4	mRNA-FC	2.6 ***			↑
Wang/2012	C-GL	3A4	Protein-FC	IND^#^			↑
Wang/2012	C-GL	3A4	PXR-3A4-FC	5 *			↑
Zhang/2022	C-GL	3A4	PXR-3A4-FC	0.285 *	↓		
He/2009	C-LQ	3A4	IC_50_	>50 μM		•	
Li/2023	C-LQ	3A4	mRNA-FC	1.64 *			↑
Li/2023	C-LQ	3A4	Protein-FC	1.58 **			↑
Xia/2012	C-LQ	3A4	PXR-3A4-FC	NE		•	
Zhang/2022	C-LQ	3A4	PXR-3A4-FC	0.459 **	↓		
Chen/2023	E-EtOH	3A4	RA-R%	NE		•	
Mao/2012	E-EtOH60	3A4	RA%	>80		•	
Mao/2012	E-EtOH95	3A4	IC_50_	15.8 μg/mL	↓		
Guo/2014	E-H2O	3A4	IC_50_	28.13 mg/mL	↓		
Sevior/2010	E-H2O	3A4	IC_50_	>100 μg/mL		•	
Guo/2014	E-H2O	3A4	RA%	67.9	↓		
Sevior/2010	E-MeOH	3A4	IC_50_	3.6 μg/mL	↓		

Huang Qi							
Author/Year	Extract/Compound	CYP	Marker/Test	Outcome	INH	NE	IND
Zhang/2017	C-ASIV	2C9	IR%	NE		•	
Xia/2012	C-ASIV	3A4	PXR-3A4-FC	NE		•	
Lau/2013	E-EtOH	3A4	IC_50_	61	↓		
Lau/2013	E-EtOH	3A4	PXR-3A4-FC	NE		•	
Mao/2012	E-EtOH	3A4	RA%	>90		•	
Zhang/2013	E-GR	3A4	CAR-3A4-FC	NE		•	
Zhang/2013	E-GR	3A4	PXR-3A4-FC	1.58 *			↑
Zhang/2013	E-INJ	3A4	CAR-3A4-FC	NE		•	
Zhang/2013	E-INJ	3A4	PXR-3A4-FC	1.6 *			↑

Bai Zhu							
Author/Year	Extract/Compound	CYP	Marker/Test	Outcome	INH	NE	IND
Dong/2008	E-H2O	3A4	PXR-3A4-FC	4.48 **			↑

Dang Gui							
Author/Year	Extract/Compound	CYP	Marker/Test	Outcome	INH	NE	IND
Sevior/2010	E-MeOH	2B6	IC_50_	11.4	↓		
Sevior/2010	E-MeOH	2B6	IR%	>80	↓		
Sevior/2010	E-MeOH	2C9	IC_50_	>100		•	
Sevior/2010	E-MeOH	2C9	IR%	NE^#^		•	
Sevior/2010	E-MeOH	2C19	IC_50_	13.7–14.3	↓		
Sevior/2010	E-MeOH	2C19	IR%	>80	↓		
Sevior/2010	E-MeOH	2D6	IC_50_	>100		•	
Sevior/2010	E-MeOH	2D6	IR%	NE^#^		•	
Li/2020	C-FA	3A4	Protein-FC	63.86 *	↓		
Li/2020	C-FA	3A4	RA%	INH **^#^	↓		
Mao/2012	E-EtOH	3A4	RA%	>100		•	
Li/2020	E-H2O	3A4	Protein-FC	115.49			↑
Li/2020	E-H2O	3A4	RA%	IND^#^			↑
Sevior/2010	E-MeOH	3A4	IC_50_	>100		•	
Sevior/2010	E-MeOH	3A4	IR%	NE^#^		•	

Chuan Xiong							
Author/Year	Extract/Compound	CYP	Marker/Test	Outcome	INH	NE	IND
Li/2020	C-FA	3A4	Protein-FC	63.86 *	↓		
Li/2020	C-FA	3A4	RA%	INH	↓		
Li/2020	E-H2O	3A4	Protein-FC	142.35			↑
Li/2020	E-H2O	3A4	RA%	IND			↑

Abbreviations: Extract/Compound (E-/C-): Compound – ASIV, (Astragaloside IV); CT, (Cryptotanshinone); FA, (Ferulic acid); GA, (Glycyrrhetinic acid); GL, (Glycyrrhizin); LQ, (Liquiritin); SAB, (Salvianolic acid B); TI, (Tanshinone I); TIIA, (Tanshinone IIA); Extract – EtOH, (Ethanolic Extract); EtOH95, (extracted by 95% ethanol); EtOH60, (extracted by 60% ethanol); GR, (Granules); H2O, (Aqueous Extract); INJ, (Injection); MeOH, (Methanolic Extract).

Marker/Test: CAR-3A4-FC, (CAR-mediated CYP3A4 activation, fold change); IC_50_, (Half maximal inhibitory concentration); IR%, (Inhibition rate, %); Ki, (Inhibition constant); mRNA-FC, (mRNA expression, fold change); PXR-3A4-FC, (PXR-mediated CYP3A4 activation, fold change); Protein-FC, (Protein expression, fold change); RA% (Relative Activity, %), RA-R%, (Remaining Activity, %).

Outcome: # (Data not reported), * (p < 0.05), ** (p < 0.01), *** (p < 0.001), IND (enhanced activity or induction observed), INH (inhibition observed), NE (no effect observed), values with units (e.g., μM, μg/mL, mg/mL) – Concentration or measured outcome as reported.

**TABLE 6:** Number of assays showing induction, No effect, or inhibition in eligible studies.

Herb	PXR	CAR	CYP2B6	CYP2C9	CYP2C19	CYP2D6	CYP3A4
Dan Shen							
Induction	3	2	‐	‐	‐	‐	4
No Effect	1	2	‐	8	8	3	8
Inhibition	‐		‐	1	1	2	6
Gan Cao							
Induction	1		‐	2	‐	‐	4
No Effect	1		1	2	2	4	4
Inhibition	2		2	3	3	1	6
Huang Qi							
Induction	2		‐	‐	‐	‐	‐
No Effect	2	2	‐	1	‐	‐	1
Inhibition	‐		‐	‐	‐	‐	1
Bai Zhu							
Induction	1		‐	‐	‐	‐	‐
No Effect	‐		‐	‐	‐	‐	‐
Inhibition	‐		‐	‐	‐	‐	‐
Dang Gui							
Induction	‐		‐	‐	‐	‐	2
No Effect	‐		‐	2	‐	2	3
Inhibition	‐		2	‐	2	‐	2
Chuan Xiong							
Induction	‐		‐	‐	‐	‐	2
No Effect	‐		‐	‐	‐	‐	‐
Inhibition	‐		‐	‐	‐	‐	2

**FIGURE 4:**
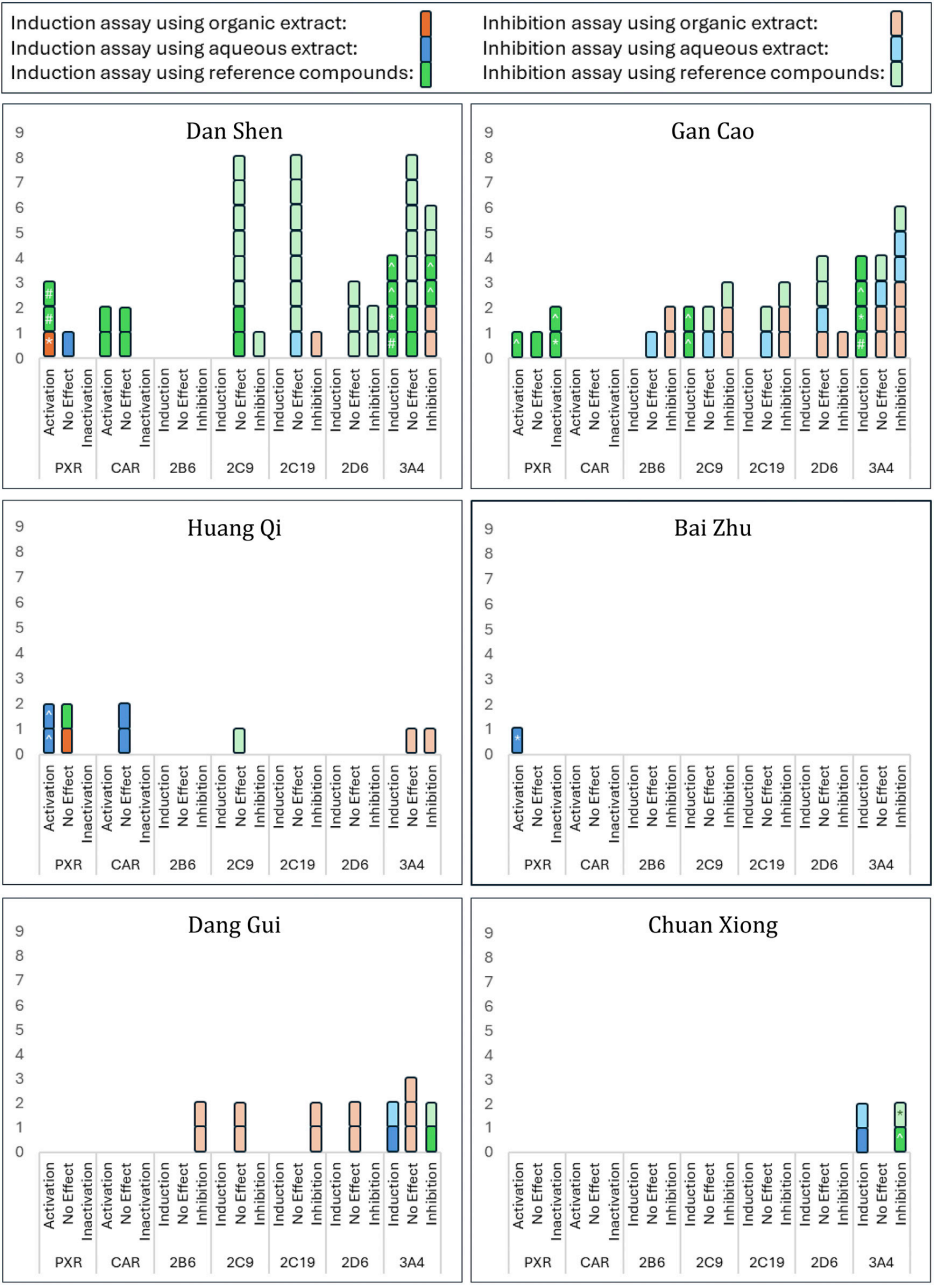
Activation/Inactivation of PXR/CAR and Modulation of CYP Enzymes by Selected Herbs. This figure summarizes findings from 24 studies on the selected herbs’ effects on PXR/CAR activation and CYP enzyme activity. Results are classified as activation, inhibition, or no effect for each enzyme. Intervention types, including different extracts and components, are color-coded as shown in the key at the top of the figure: brighter colours indicate induction assays, lighter colours indicate inhibition assays. ‘No Effect’ indicates absence of activation, induction, or inhibition. Statistical significance *versus* control, where reported, is denoted as follows: ^ p < 0.05, *p < 0.01, and #p < 0.001.

Given the diversity of methodologies and outcomes, we focused on activity trends—induction/activation, no effect, or inhibition—as the primary outcome of this review. Grouped results are displayed in Figure 4.

Notably, several studies—including Wang et al. (2011), Wang et al. (2015), Zhang et al. (2022) and Li et al. (2020) —used cell-based induction assays to measure CYP3A4 or PXR mRNA and protein expression but found decreased expression compared to controls (Table 5), indicating inhibitory effects despite the induction assay design. This finding broadens the conventional application of enzyme induction assays—typically used to detect upregulation—by demonstrating their potential utility in identifying inhibitory effects as well.

**FIGURE 5:**
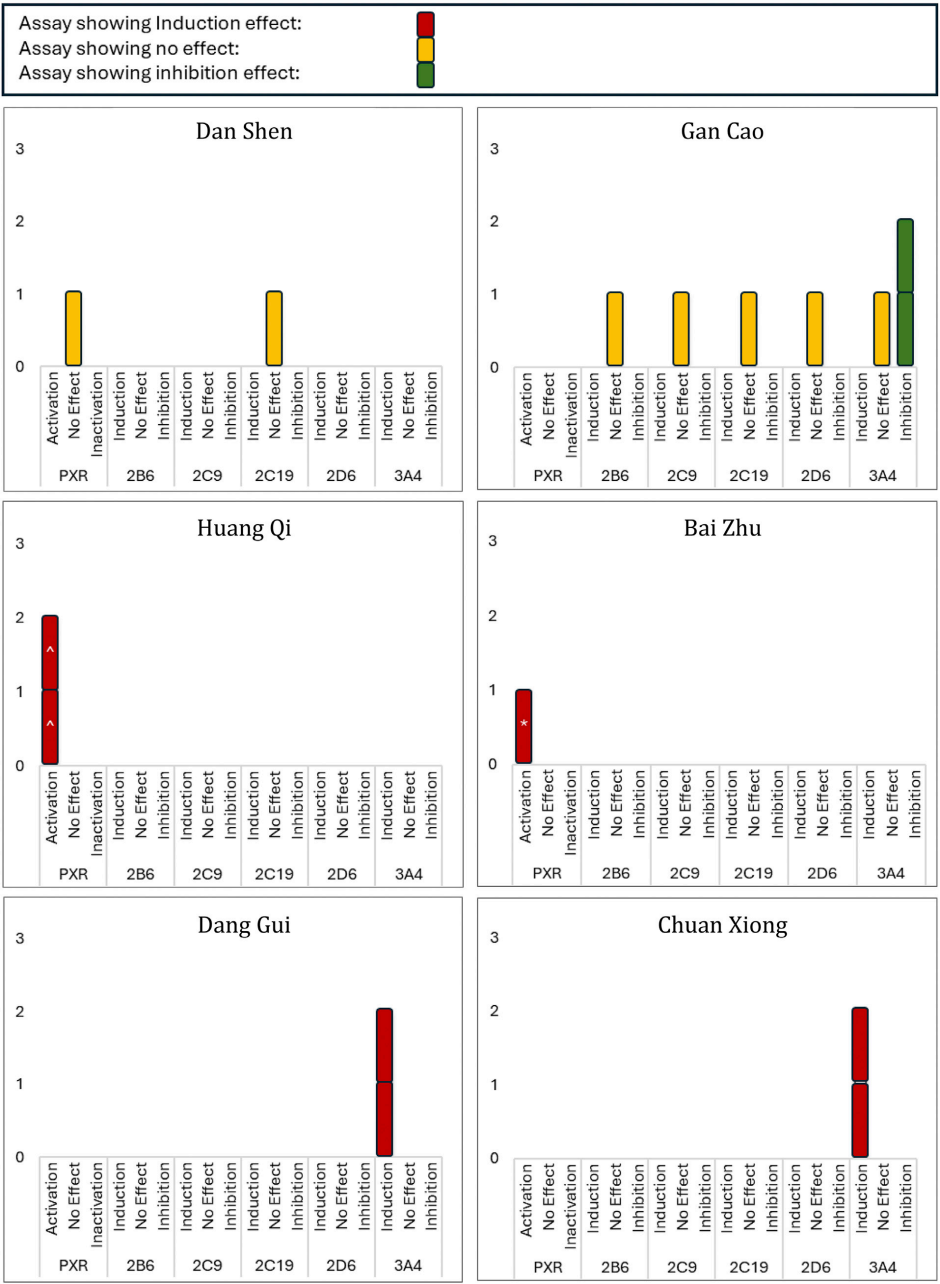
Modulation of PXR and CYP Enzymes by Aqueous Extracts of Selected Herbs. This figure presents an overview of PXR activation/inactivation and modulation of selected CYP enzymes by aqueous extracts of various herbs. Study outcomes are classified as induction, inhibition, or no effect, with color-coding to distinguish categories. ‘No Effect’ indicates no observed activation, induction, or inhibition. Statistical significance *versus* control is shown as follows: ^ p < 0.05, *p < 0.01.

Considering that aqueous extracts are the most used form of Chinese herbal medicine in clinical practice, we chose to focus on these extracts for a more clinically relevant analysis. The data from studies that used aqueous extracts to examine the modulation of PXR/CAR activation or CYPs induction/inhibition are summarized in Figure 5. Notably, all herbs, except Dan Shen and Gan Cao, demonstrated either PXR activation or CYP3A4 induction. While Dan Shen showed no effect on PXR activation, Gan Cao presented a distinct profile: two of three studies reported its inhibitory effect on CYP3A4, and one showed no effect.

### 3.5.1 Additional findings

Induction assays: Cryptotanshinone strongly increased PXR activation and CYP3A4 expression (up to 7.3-fold), while Tanshinone IIA produced moderate induction (2–4-fold). Aqueous extracts of Dan Shen showed no enzyme activation, suggesting a safer profile, whereas its ethanolic extract activated PXR (2.6-fold). Glycyrrhizin markedly activated PXR–CYP3A4 (3–5-fold), while Liquiritin induced CYP2C9 and CYP3A4 to a lesser degree (1.24–1.64-fold). Huang Qi showed minimal activation, whereas aqueous Bai Zhu extract enhanced PXR–CYP3A4 activation (4.48-fold). Both aqueous Chuan Xiong and Dang Gui extracts significantly induced CYP3A4 protein expression, increasing by 142.35% and 115.49%, respectively.

Inhibition assays: Ethanolic extracts of Dan Shen moderately inhibited CYP2C19 and CYP3A4 through mixed-type or non-competitive inhibition. Cryptotanshinone and Tanshinone I inhibited CYP2C9 and CYP3A4 competitively, while Tanshinone IIA also inhibited CYP2D6. Gan Cao methanolic extracts showed broad inhibitory effects, whereas aqueous extracts produced weaker inhibition. Glycyrrhetinic acid competitively inhibited CYP3A4 and CYP2C19, while Liquiritin had low interaction potential, as reflected by high IC50 values.

Ethanolic Huang Qi extract mildly inhibited CYP3A4, whereas Astragaloside IV showed no effect on CYP2C9. Aqueous Chuan Xiong and Dang Gui extracts enhanced CYP3A4 activity, although Ferulic Acid from these herbs exhibited inhibitory effects. Methanolic Dang Gui extracts strongly inhibited CYP2B6 and CYP2C19 but had moderate or negligible activity against other enzymes at higher concentrations.

Full experimental details and numerical data for these assays are summarized in Supplementary Tables 8.1–8.3.

## 4 Discussion

This review synthesized laboratory evidence on herb–drug interactions relevant to Chinese herbal medicines used in cardiovascular disease management and ARV therapy in people with HIV. The findings highlight both clinical opportunities and safety considerations, with implications shaped by preparation methods, cell models, and methodological quality. Key themes discussed below include clinical relevance of interaction findings, differences between extraction forms, broader safety considerations, methodological limitations, and evidence gaps requiring further research to support safe integration of Chinese herbal medicine in HIV care.

### 4.1 Clinical relevance of herb–drug interaction findings

This systematic review represents the first analysis of laboratory studies examining the effects of six Chinese herbs, commonly used in CVD management, on enzymes critical for the metabolism of ARV drugs. The quality of the studies included in this review is characterized as being of high standard and relevant, ensuring robust findings.

Herbs exhibiting minimal modulation of metabolizing enzymes may be considered safe for inclusion in herbal formulas alongside ARV therapy. However, the presence of enzyme induction or inhibition does not inherently contraindicate clinical use. In Chinese herbal medicine practice, harmonizing agents such as Gan Cao are usually employed to offset potential toxicities and regulate the formula’s overall pharmacodynamic behaviour. Thus, herbs with modulatory activity can still be used judiciously, provided they are integrated within a well-structured prescription. Importantly, when modulation is identified in one or more herbal components, the interaction potential should be evaluated at the level of the entire formula—rather than in isolation—to reflect real-world clinical use.

Each of the six herbs reviewed possesses distinct phytochemical constituents that underpin both their cardiovascular benefits and their potential to influence drug metabolism. In traditional Chinese medicine practice, herbal formulations are prescribed according to individual patient patterns (syndromes), meaning the composition and dosage of herbs may vary to address specific conditions. This individualized approach, while central to clinical efficacy, makes safeguarding against potential herb–drug interactions particularly challenging when additional herbs are combined within a formula.

### 4.2 Influence of extraction methods and preparation forms

Many studies have investigated mechanisms of enzyme modulation using isolated compounds or employed alcoholic extractions to enrich lipophilic active components for research purposes, such approaches do not fully reflect real-world clinical practice. Clinically, classical formulations contain fixed herbal products and standardized amounts of herbs, which are most commonly administered as aqueous decoctions. In this preparation, herbs are soaked and boiled to extract both hydrophilic and certain lipophilic compounds.

Notably, our synthesis highlights crucial differences between both individual herbs and their preparations. For example, aqueous extracts of Dan Shen demonstrated consistently low induction and inhibition potential across assays, suggesting a lower risk of pharmacokinetic interactions when used in traditional decoction forms. In contrast, organic solvent extracts, such as ethanolic or methanolic forms, generally showed greater enzyme modulation than aqueous extracts, potentially exaggerating interaction risks relative to typical clinical preparations. These differences underscore the importance of selecting clinically relevant extract types for research, and of evaluating each herb’s modulation profile when designing safe, effective multi-herb formulas for people with HIV receiving ARV therapy.

### 4.3 Safety considerations beyond enzyme modulation

Another important safety consideration is the potential for adverse events independent of interactions with other medications. Chinese herbal medicine can elicit side effects, however, most of which are mild and self-limiting, such as dizziness, abdominal discomfort, decreased appetite, itching, and, rarely, convulsions (Antonelli and Donelli, 2024; Cheng, 2007). In contrast, conventional cardiovascular therapies—such as statins, frequently prescribed to manage dyslipidemia in people with HIV—are well known to cause muscle-related symptoms, liver enzyme elevations, and gastrointestinal disturbances in some patients (Law and Rudnicka, 2006; Newman et al., 2019). For people with HIV who are intolerant to, or at higher risk of adverse effects from, conventional medications, Chinese herbal medicine may offer a complementary or alternative therapeutic approach.

### 4.4 Methodological issues in current evidence base

The HepG2 cell line remains one of the most employed models for studying hepatic drug metabolism and potential interactions with herbal compounds, due to its ease of use and stable characteristics. Previous studies, such as that by Wilkening et al., have shown that the induction patterns of CYP enzymes in HepG2 cells resemble those observed in primary human hepatocytes, making them suitable for preliminary screening of xenobiotic interactions (Wilkening et al., 2003). However, a notable limitation of HepG2 cells is their relatively low basal activity of several key CYP isoforms, which may restrict their utility in more detailed pharmacokinetic investigations.

To address these limitations, HepaRG cells have emerged as a more physiologically relevant hepatic model (Guillouzo et al., 2007). They more closely mimic primary human hepatocytes in both morphology and metabolic function, demonstrating higher baseline CYPs activity and broader expression of nuclear receptors and drug transporters. Future studies investigating CYP-mediated interactions between herbal medicines and ARV drugs would greatly benefit from the use of this model.

While HepG2 cells are widely used for assessing enzyme modulation, some studies (Lau et al., 2013; Li et al., 2020; Xia et al., 2012; Yu et al., 2009; Zhang et al., 2013) have selected non-hepatic cell lines as their experimental platform for various reason. These alternatives may lack the necessary enzyme expression and physiological context, potentially leading to misleading results. However, such experiments can still be considered valid if they incorporate appropriate positive controls, ensuring that the findings can be interpreted reliably despite the limitations of the non-hepatic models.

A comparative review of experimental protocols across the included studies revealed several methodological inconsistencies that may compromise the robustness and comparability of the compiled data. For instance, one study (Chen et al., 2023) used an unusually high methanol concentration (30%) in its assay, which could have confounding effects on cellular responses. Another study (Guo et al., 2014) reported IC50 values for CYP3A4 activity using an extract concentration expressed in mg/mL—substantially higher than standardized doses employed elsewhere. Furthermore, two studies (Sevior et al., 2010; Zhang et al., 2017) omitted vehicle controls, while three (Sevior et al., 2010; Xu et al., 2015; Zhang et al., 2017) lacked a positive control group, raising concerns about experimental validity and reproducibility.

Methodologically, accurate evaluation of herb–drug interactions require distinct but complementary strategies for studying enzyme induction and inhibition. Although both induction and inhibition activities can technically be assessed at the transcriptional and translational levels, as reported by Li et al. (Li et al., 2020), these markers do not reliably capture enzymatic inhibition, which more commonly occurs at the post-translational level.

Given the practical challenges of conducting parallel induction and inhibition assays for large numbers of Chinese herbal extracts, a transcription/translation-focused assay may serve as a pragmatic screening strategy. By measuring the mRNA and protein expression levels of key CYPs and their regulatory nuclear receptors (e.g., PXR, CAR), researchers can identify herbal products with the potential to alter hepatic metabolism. This enables cost-effective prioritization of herbal products for further mechanistic investigation and provides a feasible pathway for identifying low-risk Chinese herbal products.

### 4.5 Limitations of current evidence

Studying the modulatory effects of isolated herbal components provides valuable mechanistic insights into enzyme and receptor interactions. However, relying solely on component-based *in vitro* studies limits clinical applicability. Traditional Chinese herbal medicines are complex, multi-component preparations that may act synergistically, additively, or antagonistically. Findings from single compounds may therefore not accurately reflect the pharmacological behaviour of whole extracts or multi-herb formulas.

Differences in extraction methods further complicate interpretation. Ethanolic extracts, while potent and sometimes used in topical or acute treatments, differ markedly from aqueous decoctions that are standard for internal use in clinical practice. Aqueous extracts, rich in water-soluble compounds, typically offer a more gradual and sustained therapeutic effect. Variability in solvent choice, concentration, and preparation across studies may therefore influence phytochemical composition and interaction potential.

Another limitation is the narrow focus on Phase I (CYP-mediated) metabolism. Phase II pathways, including glucuronidation by UDP-glucuronosyltransferases (UGTs), sulfation, and glutathione conjugation, also play important roles in ARV drug disposition. This is particularly relevant for long-acting agents such as lenacapavir, which is primarily metabolized by UGT1A1 (Nhean et al., 2021). As these therapies become more widely adopted, evaluating both Phase I and Phase II pathways will be essential to better characterize the herb–drug interaction potential of Chinese herbal medicines with ARVs.

Additionally, the absence of *in vivo* data and clinical studies limits the translation of *in vitro* findings to real-world contexts. Finally, this review does not address the complex pharmacodynamics of multi-herb prescriptions, where constituent interactions may alter overall safety and efficacy.

### 4.6 Future directions for integrative HIV care

Herb–drug interactions remain a significant concern in HIV care. Despite growing interest in Chinese herbal medicine for conditions such as cardiovascular disease, the lack of robust pharmacokinetic and pharmacodynamic data continues to limit clinical adoption. This evidence gap fuels caution among clinicians and contributes to the exclusion of potentially beneficial therapies.

Future work should prioritize the development of an evidence-based framework for screening herb–ARV interactions, with the aim of identifying herbs that present minimal risk of pharmacokinetic interference. Establishing a list of “low risk” herbs could build confidence among clinicians and support safer integration of Chinese herbal medicine into HIV care.

This review demonstrates the methodological feasibility of using *in vitro* enzyme modulation assays as an initial screening tool. While our analysis focused on six cardiovascular-related herbs, these models are scalable and could be applied to a wider range of commonly used Chinese herbal medicines. Importantly, such studies should employ clinically relevant aqueous extracts that better reflect real-world use.

Ultimately, confirmation of *in vitro* findings through pharmacokinetic studies and clinical trials will be essential. These steps are necessary to accurately characterize interaction risks, establish safe dosing strategies, and enable evidence-based integration of Chinese herbal medicine alongside ARV therapy. Bridging traditional knowledge with biomedical safety standards will strengthen person-centred integrative care for people with HIV.

## 5 Conclusions and perspectives

This review provides the first systematic analysis of *in vitro* evidence on cardioprotective Chinese herbs and their potential to influence CYP-mediated metabolism of ARV drugs. Findings support the relative safety of Dan Shen under studied conditions, while Gan Cao shows modulatory effects consistent with its traditional harmonizing role. The results highlight the need to study aqueous whole-formula extracts, which better reflect clinical practice and capture the synergistic modulation effects of multi-herb prescriptions.

This review also demonstrates the feasibility of *in vitro* enzyme modulation assays—particularly transcriptional and translational analyses in hepatic cell lines (e.g., HepG2, HepaRG)—as scalable screening tools to identify potential herb–ARV interactions. Although we focused on six cardiovascular-related herbs, this approach can be extended to other Chinese herbal medicines commonly used by people with HIV, including those for immune support, gastrointestinal function, and antiviral effects. Broader evaluation will be essential to map the herb–drug interaction landscape more comprehensively.

### 5.1 Clinician-Focused Safety Implications

Chinese medicine practitioners should remain cautious when combining herbs with ARV therapy, particularly with CYP-modulating herbs. HIV clinicians should monitor for changes in ARV pharmacokinetics when patients use Chinese herbal medicines. The development of long-acting injectable ARVs may reduce interaction risks, but further studies are required to confirm their safety in combination with concomitant herbal use in this context.

## Data Availability

The original contributions presented in the study are included in the article/Supplementary Material, further inquiries can be directed to the corresponding authors.
